# Revealing the Properties of Plant Defensins through Dynamics

**DOI:** 10.3390/molecules180911311

**Published:** 2013-09-13

**Authors:** Ana Paula Valente, Viviane Silva de Paula, Fabio C. L. Almeida

**Affiliations:** Centro Nacional de Ressonância Magnética Nuclear de Macromoléculas, Instituto de Bioquímica Médica, Universidade Federal do Rio de Janeiro, Rio de Janeiro, RJ 21941-902, Brazil; E-Mails: vpaula@cnrmn.bioqmed.ufrj.br (V.S.P.); falmeida@cnrmn.bioqmed.ufrj.br (F.C.L.A.)

**Keywords:** defensins, dynamics, NMR, membrane recognition

## Abstract

Defensins are potent, ancient natural antibiotics that are present in organisms ranging from lower organisms to humans. Although the structures of several defensins have been well characterized, the dynamics of only a few have been studied. This review discusses the diverse dynamics of two plant defensins for which the structure and dynamics have been characterized, both in the free state and in the presence of target membranes. Multiple motions are observed in loops and in secondary structure elements and may be related to twisting or breathing of the α-helix and β-sheet. This complex behavior is altered in the presence of an interface and is responsive to the presence of the putative target. The stages of membrane recognition and disruption can be mapped over a large time scale range, demonstrating that defensins in solution exist as an ensemble of different conformations, a subset of which is selected upon membrane binding. Therefore, studies on the dynamics have revealed that defensins interact with membranes through a mechanism of conformational selection.

## 1. Introduction

Over millions of years, the major forces driving natural selection and evolution have been related to the interactions between proteins and other molecules through which the proteins perform their functions. All biological processes involve molecular recognition, which requires not only well-structured molecules capable of performing specific tasks but also dynamic molecules capable of interacting with multiple targets.

For quite some time, these binding processes were interpreted as “lock and key” recognition events in which the conformation in solution is optimized for binding and perfect specificity [1]. The ability to bind to multiple targets led to the idea of an “induced fit” process in which accommodation occurs upon binding [2]. Researchers soon recognized that these behaviors are complex and required a new approach. In the 1960s, two groups proposed the conformational selection theory, which proposed that the bound conformation was already present in solution [3,4]. Therefore, the free and bound conformations coexist in an equilibrium ensemble together with other conformational states in different proportions [5,6,7,8]. This theory has recently received much attention due to significant improvements in our ability to measure discrete states in solution. Two techniques in particular have significantly contributed to the characterization of the complexity of binding processes: nuclear magnetic resonance (NMR) and single-molecule experiments [5,8,9]. Currently, the most widely accepted theory is currently that conformational selection is the dominant binding process and occurs in conjunction with structural accommodation (“induced fit”) to optimize the interaction [10,11].

### 1.1. High-Energy States in Solution

The analysis of time- or ensemble-averaged structures does not completely describe protein function and behavior. Such a complete understanding requires not only high-resolution protein structures but also information on how these structures change over time [12,13,14,15]. Several studies have indicated that proteins do not exist as a single structure in solution but rather fluctuate among an ensemble of conformations and that these dynamic properties are essential for biological function [7,12,16,17,18,19]. This dynamism represents the balance between the tendency to occupy the lowest energy level and the tendency to populate as many levels as possible for a given level of thermal energy.

**Figure 1:**
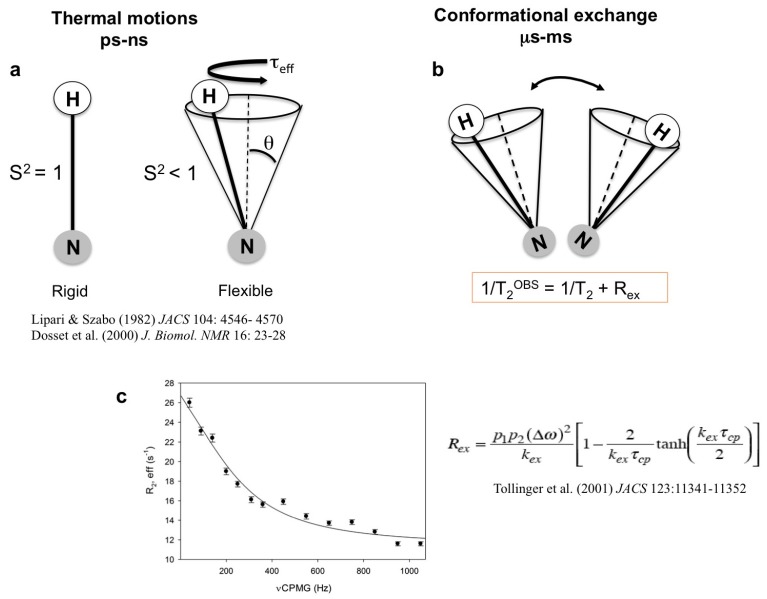
The characterization of pico- to millisecond timescales using spin relaxation measurements can probe internal motions of the bond vector. Model-free analysis of relaxation data (longitudinal relaxation (R_1_) and transverse relaxation (R_2_)) yield an order parameter (S^2^) that describes the amplitude of motion, a constant (τ_eff_) describing its timescale [37,38,39] (**a**) and R_ex_ that describes the additional exchange contribution in R_2_ (**b**). A representative relaxation dispersion curve demonstrating the dependence of R_ex_ that can be used to extract the populations (p), exchange rates (k) and chemical shifts (ω) of ground and exited states (see the equation) [40]. The curve was fitted using CPMGFit program (www.palmer.hs.columbia.edu) [41] (**c**).

Discrete conformational states are separated by energy barriers that are related to the kinetics of interconversion [5,7]. Proteins interconvert between states over a broad range of time scales, from picoseconds to nanoseconds for bond oscillations and rotations, from micro- to milliseconds for loop and larger domain motions and from seconds to hours for slower motions such as proline isomerization [19,20]. NMR spectroscopy is able to probe a broad range of time scales, and different strategies can be used to quantify each type of motion (see Figure 1 and Appendix 1) [19,20,21,22,23,24,25,26,27,28,29]. This methodology can provide quantitative information on intermediates and other excited states that are sparsely populated and cannot be observed by conventional methods [14,15,16,17,18,19,20,21,22,23,24,25,26,27,28,29,30].

Important results have been obtained using a relaxation dispersion technique that permits the analysis of conformational exchange on the µs-ms time scale [24,25,31]. Kern and co-workers analyzed adenylate kinase and demonstrated that the opening of the nucleotide lid is the rate-limiting step for enzymatic catalysis [32,33,34,35]. Additionally, Kay and co-workers analyzed the T4 lysozyme mutant L99A and demonstrated that it interconverts between a ligand-inaccessible conformer and a ligand-accessible state at a 2 kcal mol^−1^ higher free energy, which is referred to as the excited state. The excited state is characterized by a decrease in the number of stabilizing interactions relative to the ground state, with an increase in entropy that is consistent with a more dynamic structure [36].

The structural details of the excited state have recently been obtained using an innovative strategy applying NMR and chemical shift calculation [30]. The proposed strategy has been shown to be robust and has already been used to analyze several proteins [42].

One new challenge in this area is the effort to identify the structural elements that are responsible for dynamics, which can be considered important “errors” that maintain a dynamic the protein structure and therefore prompt interaction. A perfectly stable, structured protein can be imagined to be as unable to interact with other proteins or molecules. In this sense, it is important to identify protein segments involved in the conformational equilibrium that can exchange their energy content between different regions of the protein [43]. Using a numerical surface cooling approach, Piazza and Sanejouand suggested that dynamic protein segments can exchange kinetic energy during conformational changes [44,45]. These authors demonstrated that specific regions of the protein structure can store energy that amino acids residues involved in catalysis tend to be located in such regions. Anisotropic energy flows through the vibrational states of proteins. Globular proteins contain channels for the propagation of energy depending on the density of the vibrational states [46].

This review will focus on the application of NMR methods to study the interactions between defensins and a biological membrane. Relaxation measurements have revealed that in membrane interaction events, protein binding sites exhibit conformational equilibria among a pre-existing array of conformations. In the presence of a ligand, one conformation is stabilized followed by a population shift toward the bound conformational state [8].

### 1.2. Plant Defensins

Defensins are a large family of host defense peptides that are widely distributed with more than one thousand identified in plants, insects, birds, fish and mammals. Defensins exhibit a wide range of functions that include antimicrobial action (against fungi, bacteria and viruses), chemotaxis and signaling. Human β-defensins, in addition to the antimicrobial activity, are also involved in linking the innate and adaptive immune systems [47,48,49,50,51].

The three-dimensional structure of β-defensins is well characterized and several proteins have been studied using X-ray crystallography or NMR. A structural comparison indicates that although β-defensins exhibit a diverse amino acid composition, the cysteine-stabilized α/β fold is conserved. The arrangement produces a thermally stable structure that is affected by changes in the primary sequence and is highly evolvable. The defensin scaffold is common for several activities, including antifungal, antibacterial and ion channel blockers activity and the inhibition of α-amylase [52,53,54]. Although highly conserved the disulfide bonds are not essential for antimicrobial function as observed for human β-defensin 3, θ-defensin [55,56].

We have studied the dynamic properties of defensins and their interactions with model membrane systems. Plant defensins are small (45–54 amino acids), highly basic, cysteine-rich peptides that are structurally related to the defensins of other organisms, including insects and mammals.

Despite the extreme primary sequence diversity among defensins, the following important structural features can be identified by comparing various plant defensins: (a) defensins exhibit a high level of diversity in their primary structure despite possessing an identical overall fold that is composed of three antiparallel β strands, one α-helix and four disulfide bonds; (b) lack of amino acid signature that account for the diverse array of activities, which hinders the prediction of antimicrobial activities; (c) defensins exhibit host-pathogen co-evolution in which the ability to accommodate mutations is an important tool in avoiding resistance; (d) defensins are positively charged at physiological pH, and this positive charge facilitates the initial interactions with the anionic head groups of microbial membrane lipids; and (e) defensins possess an amphipathic character that enables their interactions with the core of the membrane. Recent studies have indicated that damage to the membrane is only one of several mechanisms involved in the antibiotic action of defensins [57,58].

Defensins exhibit a diverse amino acid composition, whereas the structure-stabilizing cysteines appear to be conserved. The α/β arrangement produces a thermally stable structure that is affected by changes in the primary sequence and is highly evolvable.

Substantial efforts have been made to correlate the structure of defensins with their function; however, the complexity of the system has hindered the identification of a structural signature for each activity [59,60]. Yount and Yeaman have proposed a γ-core motif (GXCX_3–9_C) that is formed by the β2 and β3 strands and the loop between them. The presence of this highly conserved motif has been recognized as a hallmark of defensins that demonstrate antimicrobial activity [61,62]. Recently, Sagaram *et al*. demonstrated that synthetic γ-core variants of MtDef4 induced plasma membrane permeabilization. According to these authors, although the γ-core motif exhibits antimicrobial activity, the full-lenght protein is more efficient; this finding suggests that not only the primary sequence but also additional features are responsible for the antimicrobial activity of these defensins [63].

## 2. Probing Membrane Interaction

Several studies have indicated that membrane permeation or disruption is not the only mechanism involved in the antimicrobial activity of defensins, but it is the first barrier that must be overcome. Micelles and vesicles are mimetic systems that are highly useful to probe membrane recognition events. Vesicles are considered more physiologically relevant due to their low curvature and kinetic stability; however, they present challenges because for high affinity complexes, vesicles yield broad NMR signals. Although micelles possess a high curvature, they are smaller and, therefore, tumble faster, resulting in better-resolved NMR signals. Moreover, because micelles present a higher degree of hydrophobic exposure than vesicles, combining the results obtained from the two systems can provide information on initial and subsequent events in the process of membrane interaction [60].

The most difficult task is the determination of specificity. Many peptides and proteins can interact with interfaces, but this does not mean that this interaction is related to their biological functions. Therefore, our experimental systems include that sphingolipid glucosylceramide (CMH), which is a component of the fungal membrane. This component has been implicated in defensin action, as the deletion of the gene responsible for its synthesis produces a CMH-depleted fungal plasma membrane that is highly resistant to defensin action [64].

**Figure 2:**
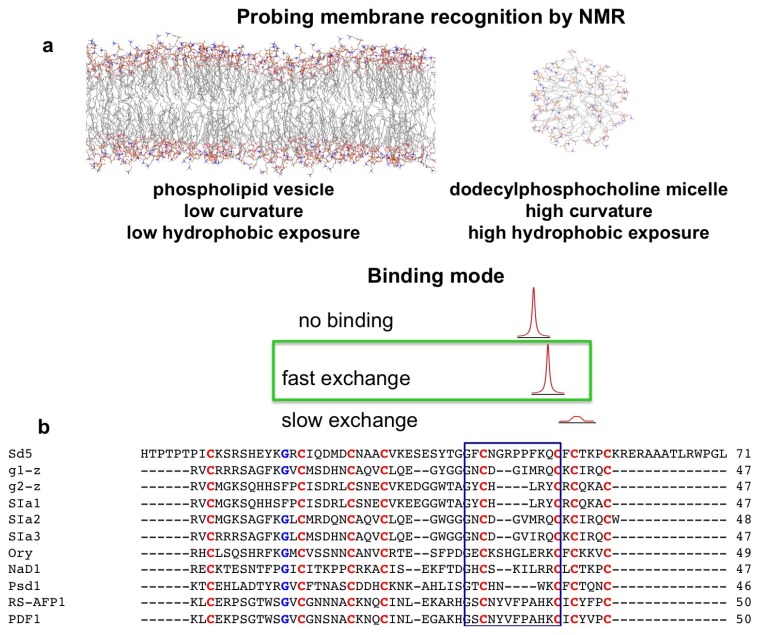
Common membrane mimetic systems and sequence alignment of plant defensins used in this study to probe events of membrane recognition. The resulting NMR spectrum of a given nucleus depends on the affinity of the membrane-defensin complex. In a fast exchange regime, the spectrum exhibits sharp lines. If the interaction is too strong (high affinity complexes), the spectrum exhibits broad NMR lines (**a**). Comparison of the primary sequence of representative plant defensins. Conserved cysteine residues are colored in red and the conserved glycine residue is highlighted in blue. The γ-core motif (GXC or CXG-C) is indicated by a blue box [61] (**b**).

Another important aspect is the mode of membrane interaction. Solution NMR studies require the interaction to occur in the fast exchange regime. If the interaction is too strong, which leads to slow exchange, the process will be dominated by the exchange between free and interface bound states rather than by the conformational exchange process per se (see Figure 2a).

Studies of protein dynamics have significantly contributed to the understanding of the relationship between the structure and function of plant defensins. To date, the structure-function relationship and dynamics have been simultaneously studied for only sugarcane and sweet pea defensins. These studies complement the structural characterization and demonstrate that proteins of similar structure can exhibit very different dynamic properties that may determine binding and specificity.

### 2.1. Sd5

To search for new defensins, six putative proteins (MW of 5–10 kDa) containing eight cysteines were screened based on the sugarcane expressed sequence tag (EST) database [65]. Structural characterization based on circular dichroism (CD) and nuclear magnetic resonance spectroscopy indicated that the structures of these Sds were compatible with those of α/β proteins.

Sd5 is the result of a relatively recent gain of function, as it exhibits similarity to only defensins in the Andropogoneae tribe and most likely evolved less than 9 million years ago [65]. The structure of Sd5 has been characterized using NMR and adopts a typical cysteine-stabilized α/β motif (CSαβ) structure, highly similar to those of other plant defensins, which is expected from the strong conservation of the defensin fold. The major structural difference is the presence of an unstructured C-terminal region in Sd5 [60].

Despite stabilization by four disulfide bonds, ^15^N relaxation rates revealed that several regions of this protein are dynamic, with at least two distinct motions that account for the conformational fluctuations: the first loop and the C-terminus exhibit motion on the ps-ns timescale, and the second loop and elements of secondary structure exhibit motions on the ms-µs timescale. Studies at different temperatures indicated that these motions range from intermediate to fast exchange.

**Figure 3:**
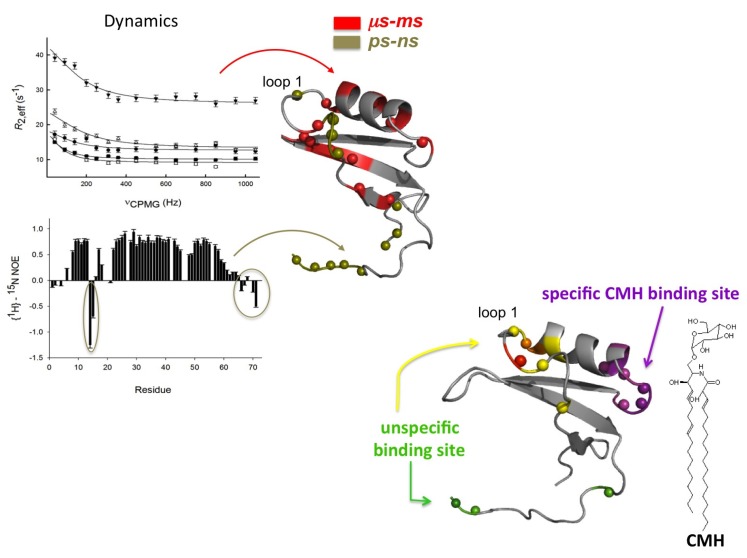
Complex motions of Sd5 probed by backbone relaxation parameters. Residues exhibiting fast motion (ps-ns) are probed using both micelles and vesicles, but those involved in conformational exchange are probed only in the presence of CMH. The regions mapped in membrane recognition with different dynamic properties are highlighted in purple, green and yellow.

We used Carr–Purcell–Meiboom–Gill (CPMG)-based dispersion experiments [66], which are sensitive to millisecond (ms) exchange dynamics between states, to quantitatively probe these motions and the results indicated that Sd5 fluctuates on this timescale (see Figure 3).

**Figure 4:**
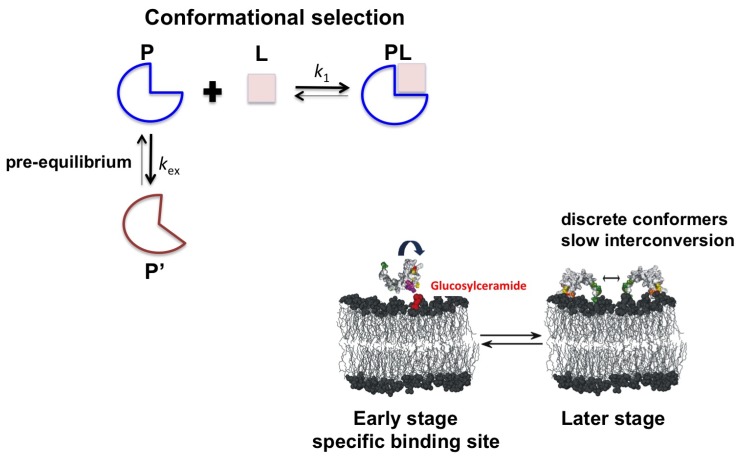
Proposed model for the Sd5 interaction surface with a membrane mimetic. In a conformational selection mechanism, the binding competent conformation (blue, P) preexistts in solution prior to the addition of the ligand (L). Residues affected by the interactions with PC:CMH vesicles are colored in purple (the specific binding site), and DPC micelles are colored in yellow and green (the nonspecific binding site). The component of the fungal membrane, glucosylceramide (CMH), is highlighted in red.

To understand the importance of such complex motions in Sd5 membrane recognition, we evaluated the effect of membrane models on the dynamic properties of Sd5 in combination with chemical shift perturbation. Residues exhibiting fast motion (ps-ns) were probed using both micelles and vesicles, but those involved in conformational exchange were probed only in the presence of CMH. Two sites were identified: a nonspecific site that participates in the recognition of the interface and a specific site that recognizes CMH. These results suggest that Sd5 interacts with the CMH interface through a conformational selection process. The binding region is in exchange between the free and the bound conformation. The interface stabilizes the bound conformational state and shifts the equilibrium toward the bound form (see Figure 4).

In the early stages of membrane interaction, which have been mapped using phospholipid vesicles, the binding sites exhibited increased conformational exchange on the µs-ms timescale, leading to increases in the R_2_ values. Residues involved in exchange in the free state were perturbed, and contiguous regions also underwent conformational exchange upon vesicle interaction. In the later stages of membrane insertion, as mapped using dodecylphosphocholine (DPC) micelles, Sd5 was stabilized in several discrete conformations exhibiting slow dynamic exchange. Our studies monitored protein dynamic properties during the events of membrane recognition and insertion and contribute to the understanding of the structural and dynamic requirements associated with defensins-membrane interaction and disruption.

Using this approach we identified two distinct regions of the Sd5 structure that exhibit different dynamic properties. The flexible regions are involved in nonspecific interaction with the interface, whereas the regions that undergo conformational exchange are involved in specific binding. In conclusion, Sd5 interacts with the CMH-membrane through a process of conformational selection (Figure 4).

### 2.2. Psd1

**Figure 5:**
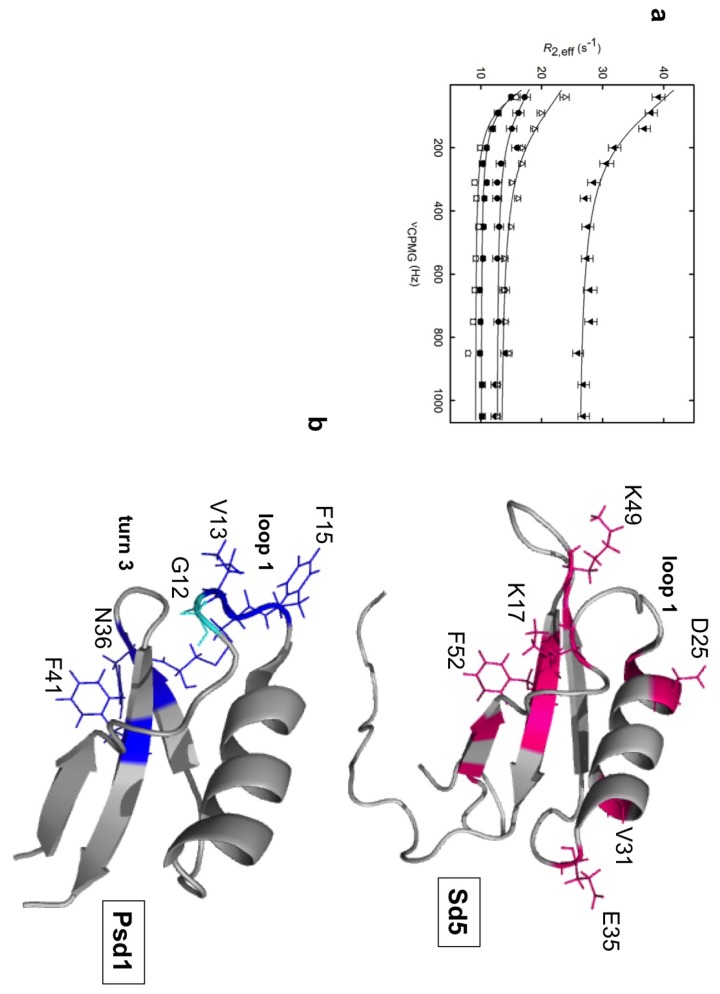
Dynamics properties of the plant defensins Sd5 and Psd1. Quantitative analysis of exchange dynamics in Sd5. ^15^N-NMR relaxation dispersion curves were measured at 800 and 600 MHz. Side chains of the residues with amide backbone conformational exchange measured by NMR relaxation dispersion are colored magenta in the NMR structure of Sd5 (PDB code 2KSK) (**a**). Ribbon representation of Psd1 highlighting the side chains of the residues in conformational exchange in blue and the flexible hinge Gly12 in cyan. Gly12 is the only residue with decreased order parameter, which indicates thermal flexibility (PDB code 1JKZ) (**b**).

The complex behavior observed for Sd5 is not shared by sweet pea defensin (Psd1). Psd1 possesses the cysteine-stabilized α/β motif, and its dynamic properties were evaluated using NMR in the presence of various membrane mimetic systems to determine the properties that are important for its antifungal activity [59,67]. When free in solution, the R_1_ and R_2_ values are similar for most residues, which is a feature that is consistent with a well-structured protein. The only exception occurs in residues 13–15, which exhibit higher than average R_2_ values, suggesting that this region undergoes conformational exchange in solution. This region lies near the conserved glycine 12 that exhibits relaxation values compatible with a flexible residue. Our interpretation is that this ps-ns timescale movement acts as a hinge, implying a µs-ms timescale exchange for the remainder of the loop. The fitting of R_ex_ either by Lipari-Szabo model-free formalism or by R_2_/R_1_ values indicates the presence of conformational variability in Loop1 and Turn3 with the exchange between conformers occurring on the micro- or milliseconds timescale (see Figure 5). The fact that the motion of Loop1 is correlated with that of Turn3 is not surprising, as they are connected by a disulfide bond (Cys14-Cys35). Figure 5 shows a comparison of residues involved in conformational exchange in both defensins Sd5 and Psd1.

Changes in the Psd1 structure and dynamics were evaluated using an approach similar to that described above. Psd1 interacts with vesicles of PC and PC:CMH, but the line shape did not significantly increase which indicates that the interaction occurs in the fast exchange regime. Similar regions were probed in both systems, and greater changes were observed in Loop1 and Helix1. As this region is a positive patch on the Psd1 surface, these results suggest an electrostatic interaction with the surface. As in the previous case, the addition of CMH promotes additional changes to those observed for PC vesicles. For Psd1, these changes occurred in Loop1 and Turn3. This identical region undergoes conformational exchange when the molecule is free in solution. These results suggest that Psd1 also interacts with the membrane through a conformational selection process.

The presence of PC:CMH vesicles induced several changes in the dynamic properties of Psd1. The R_2_/R_1_ ratio of Cys14, Phe15 and His36 decreased whereas that of residues Asn17, His29, His36, Asn37 and Trp38 increased.

We subsequently studied this interaction using micelles. Identical residues that were probed using phospholipids vesicles, and Loop1 and Turn3, were additionally monitored using DPC micelles.

Because Loop1 was identified as interacting with the interfaces, we synthesized a peptide that corresponded to the region perturbed by the presence of CMH to evaluate whether this peptide, called pepLoop1, could maintain the interaction properties in the absence of the surrounding protein structure. PepLoop1, which includes residues 7 to 17, did not exhibit a stable structure in solution but was stabilized in the presence of DPC micelles with or without CMH. Furthermore, the presence of consistent NOEs and changes in scalar couplings allowed us to evaluate the stabilized structure. The presence of CMH appears to stabilize a more extended conformation, and the specificity of this interaction was confirmed by the assignment of several unambiguous NOEs from pepLoop1 and CMH. These results demonstrate that pepLoop1 may represent the minimal domain required for the interfacial interaction of Psd1. We believe that Loop1 is responsible for the ability of Psd1 to interact with interfaces and that other regions are responsible for the regulation of Psd1 activity and/or its interactions with other targets in the cell [57].

## 3. Conclusions

Antimicrobial peptides are constantly evolving to overcome continuous environmental changes and microbial adaptation. Defensins are one of the most prevalent families of antimicrobial peptides and they share low primary sequence similarity despite possessing an identical overall fold. This sequence diversity reflects a variety of mechanisms of membrane interaction.

Silvestein *et al*. estimated that approximately ~2%–3% of plant genes code for AMPs [54]. Substantial efforts have been made to understand the structure-function relationships of membrane-destabilizing peptides with the aim of rationally designing novel molecules for biotechnological applications in the fields of antibiotics, biosensors and drug delivery.

The relation of structure to function is neither obvious nor easily predictable. The α/β defensin fold is highly robust and can tolerate mutations without a loss of biological activity, a feature that ensured its ability to evolve [68]. Evolutionary success is based on the “promiscuity” of the fold, which permits the evolution of different molecules with a similar fold. The process of decoding the structural information is challenging and includes the determination of the structure and characterization of the dynamics of the protein and changes that occur upon membrane interaction.

Defensins appear to interact with membranes through a conformational selection process because in solution they exist as structural ensembles. Future studies to understand the recognition of the membrane will include the determination of the structures of defensins in the excited state. Relaxation dispersion experiments will reveal the details of the conformers that will aid in the design of new and more efficient molecules.

## Appendix 1: The application of NMR to the study of interactions and dynamics

Solution NMR spectroscopy is well suited for the experimental characterization of the defensin-membrane interaction at the atomic level. Structural information can be obtained through direct analysis of the difference in resonance frequencies of each nucleus, which provides information on the chemical environment [59,69,70]. The ligand is added to an isotopically labeled protein, and changes in the frequencies for residues involved in intermolecular contacts are recorded. These perturbations are then mapped onto the protein structure to define binding sites.

The protein dynamics can also be probed by measuring relaxation parameters. NMR allows for the dynamics to be investigated over a wide range of time scales [71]. Measurements of ^15^N relaxation parameters can be translated into atomic information on the dynamics using the Lipari and Szabo model free formalism [37,38] and extended by Marius Clore [72]. The result of this fitting procedure is the order parameter (S^2^) of the movements that occur in the ps-ns time-scale and the k_ex_ of movements that occur in the μs-ms, which are related to conformational exchange.

The conformational exchange process can be quantitatively analyzed by performing relaxation dispersion experiments [14]. These experiments yield the rate constant of the motion that occurs on the μs-ms timescale. The global analysis of the data can provide information on the kinetic and thermodynamic parameters that describe the conformational exchange events, including the chemical shift changes between exchanging states. In several cases, the structure was successfully determined in the excited state using the obtained chemical shift values from relaxation dispersion experiments and protocols for structural calculations based on chemical shifts such as CHESHIRE [73] or CS-Rosetta [74].
